# Reliability and Validity of the Telephone-Based eHealth Literacy Scale Among Older Adults: Cross-Sectional Survey

**DOI:** 10.2196/jmir.8481

**Published:** 2017-10-26

**Authors:** Michael Stellefson, Samantha R Paige, Bethany Tennant, Julia M Alber, Beth H Chaney, Don Chaney, Suzanne Grossman

**Affiliations:** ^1^ Department of Health Education and Promotion East Carolina University Greenville, NC United States; ^2^ Department of Health Education & Behavior University of Florida Gainesville, FL United States; ^3^ ICF Fairfax, VA United States; ^4^ Kinesiology Department California Polytechnic State University San Luis Obispo, CA United States; ^5^ Department of Community Health and Prevention Drexel University Philadelphia, PA United States

**Keywords:** social media, aging, health literacy, Web 2.0, Internet

## Abstract

**Background:**

Only a handful of studies have examined reliability and validity evidence of scores produced by the 8-item eHealth literacy Scale (eHEALS) among older adults. Older adults are generally more comfortable responding to survey items when asked by a real person rather than by completing self-administered paper-and-pencil or online questionnaires. However, no studies have explored the psychometrics of this scale when administered to older adults over the telephone.

**Objective:**

The objective of our study was to examine the reliability and internal structure of eHEALS data collected from older adults aged 50 years or older responding to items over the telephone.

**Methods:**

Respondents (N=283) completed eHEALS as part of a cross-sectional landline telephone survey. Exploratory structural equation modeling (E-SEM) analyses examined model fit of eHEALS scores with 1-, 2-, and 3-factor structures. Subsequent analyses based on the partial credit model explored the internal structure of eHEALS data.

**Results:**

Compared with 1- and 2-factor models, the 3-factor eHEALS structure showed the best global E-SEM model fit indices (root mean square error of approximation=.07; comparative fit index=1.0; Tucker-Lewis index=1.0). Nonetheless, the 3 factors were highly correlated (*r* range .36 to .65). Item analyses revealed that eHEALS items 2 through 5 were overfit to a minor degree (mean square infit/outfit values <1.0; *t* statistics less than –2.0), but the internal structure of Likert scale response options functioned as expected. Overfitting eHEALS items (2-5) displayed a similar degree of information for respondents at similar points on the latent continuum. Test information curves suggested that eHEALS may capture more information about older adults at the higher end of the latent continuum (ie, those with high eHealth literacy) than at the lower end of the continuum (ie, those with low eHealth literacy). Item reliability (value=.92) and item separation (value=11.31) estimates indicated that eHEALS responses were reliable and stable.

**Conclusions:**

Results support administering eHEALS over the telephone when surveying older adults regarding their use of the Internet for health information. eHEALS scores best captured 3 factors (or subscales) to measure eHealth literacy in older adults; however, statistically significant correlations between these 3 factors suggest an overarching unidimensional structure with 3 underlying dimensions. As older adults continue to use the Internet more frequently to find and evaluate health information, it will be important to consider modifying the original eHEALS to adequately measure societal shifts in online health information seeking among aging populations.

## Introduction

The increasing amount of online health information available to the public [1,2], coupled with the popularity of health-related Internet searches [3,4], has greatly increased Internet use for health-related purposes. With this increased use come both benefits and challenges. Greater Internet adoption has increased the availability of health information for consumers, yet disparities in access to relevant online health information persist, especially among users with insufficient skills to discriminate between credible and fraudulent online health information. The broad reach of the Internet has potential to increase health knowledge and to build self-efficacy to carry out protective health behaviors, yet the large volume of health information on the Internet often lacks quality, relevance, and veracity [5,6]. Online health information seeking is also generally an independent, goal-driven activity that puts the user in control of sifting through an abundant amount of health information. To do this effectively, users must possess skills to identify reliable sources, appraise the relevance of online health information, and translate knowledge gained into meaningful action that addresses a health-related concern.

### Older Adults and Online Health Information Seeking

Proficiency in carrying out online health information-seeking behaviors varies by sociodemographic factors, including age [7]. For example, greater adoption of the Internet by older adults has increased the accessibility of health information to this subset of the population [8,9]. One recent study in the United States showed that Web adoption among older adults is climbing, with 67% of people over 65 years of age using the Internet and more than 40% using smartphones [10]. Over 50% of US adults aged 35 to 60 years reported searching for online health information, while only 31% over the age of 60 years reported doing so [11]. Older adults need high-quality, relevant, and accurate health information regarding age-related physical conditions and ailments that require regular and consistent medical attention [12,13]. However, research suggests that most older adults do not access high-quality health information that addresses their health concerns [14].

There are several reasons why older adults may be unable to benefit from increased access to online health information. Older adult populations report high computer anxiety, which compromises their ability to carry out functional tasks using Internet-based technologies [15]. Only 26% of older adult Internet users reported feeling confident when using the Internet to complete daily tasks [10]. This lack of confidence using digital devices often leads to lack of Internet use for health information among older adult populations [10,16-18]. Nevertheless, older adults who overcome anxiety toward using health information technology demonstrate greater patient activation (ie, enhanced knowledge, skills, and confidence a person has in managing their own health and health care) and are more satisfied after talking with their provider about their own medical questions [19,20].

Moreover, routine online health information seeking has the potential to motivate older adults living with chronic disease to become more proactive in their health care decision making [21,22]. Because it is very likely that older adults will increasingly use the Internet to access health information to improve their health, it is important to measure the extent to which they have the capacity to search for, retrieve, and evaluate health-related resources that they come across online (ie, eHealth literacy).

### Measurement of eHealth Literacy

eHealth literacy was originally defined by Norman and Skinner [23] as “the ability to seek, find, understand, and appraise health information from electronic sources and apply the knowledge gained to addressing or solving a health problem.” To conceptualize eHealth literacy, Norman and Skinner [23] used the metaphor of a lily flower with 6 discrete petals (literacies) feeding into a core pistil. They categorized the core literacies proposed to contribute to eHealth literacy as being either context specific (ie, health, computer, and science literacies) or analytic specific (ie, traditional and numeracy, information, and media literacies). The concept of eHealth literacy is dynamic and evolving, meaning it varies per a variety of individual and contextual factors, including an individual’s health status, their purpose(s) for seeking health information, and the technology they select to access health information. Recent research suggests that people with greater eHealth literacy are more informed health decision makers [24], which ultimately increases their capacity to engage in health protective behaviors [25] and improve their quality of life [26]. While several studies have examined eHealth literacy, rigorous measurement of the 6 constituent eHealth literacies is underdeveloped and presents an ongoing challenge for health promotion researchers.

In 2006, Norman and Skinner [27] developed the eHealth Literacy Scale (eHEALS), an 8-item rating scale that measures consumers’ knowledge of and perceived confidence in their ability to seek, understand, and evaluate health information obtained from the Internet to address health-related concerns. Scores from eHEALS have supported its reliability as a unidimensional scale in diverse populations, including adolescents [27], college students [28], adults in the general US population [28], older adults recruited on the Internet [29], and people living with chronic disease [30,31]. eHEALS has been translated into many different languages and administered in countries around the world (eg, Germany, Italy, the Netherlands, Israel, and China).

Several studies have explored the dimensionality of data produced by eHEALS, reporting varied results. This literature describes some potential problems related to the internal structure of the eHEALS. Specifically, the number of factors (and factor loadings) derived in measurement studies of the eHEALS have shown some variability. Two recent studies reported that up to 3 unique, yet highly correlated, factors may be present when the scale is administered to older adults [30,32]. However, studies reporting the presence of multiple subscales have yet to explicate which eHEALS items load onto distinct factors (or constructs) when eHEALS is completed by older adults [29,30,32]. This variability has caused some difficulty when attempting to define what these unique factors, or subscales, are actually measuring.

Soellner and colleagues [33] translated the eHEALS into German and found that, despite poor global model fit, data from 18-year-old university students may best fit a 2-factor model, where eHEALS items measure online health information seeking (items 1-5, and 8) and online health resource appraisal (items 7 and 8). Neter and colleagues [7] also found adequate global model fit with a 2-factor model of eHEALS data when collected among adults over 21 years of age. This 2-factor structure consisted of 1 factor measuring online health information seeking (items 1-3) and another measuring online health resource appraisal (items 4-8). It should be noted, however, that 1 study [33] primarily consisted of adolescents. Younger people are more likely to report higher eHealth literacy than their older counterparts [7,31]. The factor structure and variance of eHEALS scores may differ as a function of age, which could influence results from eHEALS studies including younger versus older samples. Diviani et al [34] conducted a validation study of the Italian version of eHEALS administered among young to middle-aged adults (mean age 37.37 years, SD 13.78). Confirmatory factor analysis results showed suboptimal model fit among 2 rival models (1-factor structure vs 2-factor structure), yet parametric and nonparametric item response theory (IRT) analyses confirmed that the single-factor model best fit the data in the study sample. However, studies reporting the presence of multiple subscales have yet to explicate which eHEALS items load onto distinct factors (or constructs) when eHEALS is completed by older adults [29,30,32].

It is also important to note that the mode of survey administration can affect the reliability and validity evidence of survey data [35]. Many studies examining the internal structure of eHEALS data collected from older adults have only used Web-based survey methods [29,30,32]. Web-based surveys have several advantages, including time and cost efficiencies, but they are prone to response bias, especially when respondents demonstrate concerns about the privacy of disclosing information through Web-based survey portals [36]. Also, analyzing eHEALS data collected from only active Internet users may reduce the quality of reliability and validity assessments due to sampling bias. Older adults who use the Internet to complete the eHEALS are more likely to be more confident in their online health information-seeking skills; thus, solely relying on Web-based survey methods to establish evidence for the validity of eHEALS scores may introduce measurement bias. Administering Web-based versions of eHEALS to older Internet users may skew data toward respondents with high overall eHealth literacy, which may partly explain why existing studies report moderate to high eHealth literacy in older adult populations.

Dillman [37] recommended use of telephone-based surveys for collecting data among older populations, who often feel more comfortable answering questions asked by an actual person rather than via online or paper-and-pencil questionnaires. In a recent study, Neter and Brainin [38] conducted a nationally representative random digital dial telephone household survey of Israeli adults aged 50 years and older to determine their perceived eHealth literacy as measured by eHEALS. In this older population, perceived eHealth literacy was judged to be moderate (mean 3.17, SD 0.93), with a moderate correlation established between perceived and actual eHealth literacy (*r*=.34, *P*=.01). However, no psychometric data on eHEALS responses was reported in this age-restricted (50 years of age and older) sample. Therefore, much variability has been documented in the literature and has led to difficulty defining what the unique factors, or eHEALS subscales, may be measuring. These discrepancies in confirmatory factor analysis and IRT analysis results highlight the importance of conducting additional psychometric research that considers differences in eHEALS item measurement, factor structure, and item difficulty among older adults. The purpose of this study was to examine the reliability and explore the internal structure of eHEALS data, when the scale is administered to older adults using telephone-based survey methods.

## Methods

### Recruitment

We conducted a cross-sectional landline telephone survey as part of the Florida Consumer Confidence Index (F-CCI) Survey [39]. At least 500 households in the US state of Florida were contacted over 1 month. A minimum of 10 call attempts per household were made every Monday through Friday (between 9:00 AM and 9:00 PM), Saturday (between 12:00 PM and 6:00 PM), and Sunday (between 3:00 PM and 9:00 PM) using the random digit dialing method. The Institutional Review Board at the University of Florida approved the conduct of this study. Overall, 6695 calls were placed, and 493 individuals (response rate 7%) agreed to participate in the telephone survey. Participants were not provided incentives as part of participating in the F-CCI. We included data from these individuals in the main analyses if respondents reported being (1) at least 50 years old, and (2) Internet or email users. We selected the age cutoff based on Watkins and Xie’s [40] systematic review of eHealth literacy interventions for older adults, citing that chronological age for the older population “can range from 50 to over 100” years, and the age range of 50 years and older “is consistent with growing appreciation of the role that health behavior interventions play in healthy aging for those under age 65” years (pg e255). While screening participants for this study, we found that 393 F-CCI Survey respondents reported being at least 50 years old, yet 110 responded “no” when asked if they used the Internet or email. Therefore, the final sample size for this study was N=283.

### Measures

#### Sociodemographics and Health Status

We asked respondents to provide the following personal information: (1) age (in years); (2) sex (male, female); (3) race (white, African American, Asian or Pacific Islander, American Indian or Alaskan Native, multiracial or mixed race nonwhite); (4) ethnicity (Spanish or Hispanic, non-Spanish or non-Hispanic); (5) education (less than high school, high school or general equivalency diploma, some college, college graduate, postgraduate); (6) income (less than US $20,000, $20,000-49,999, $50,000-$99,999, $100,000 or more); and (7) perceived health status (poor, fair, good, very good, excellent). Additionally, respondents reported whether they had any experience (yes/no) using social media platforms (ie, online support group, popular social media websites such as Facebook or Twitter, or online blogs) to access or share health information.

#### eHealth Literacy

Norman and Skinner’s [27] eHEALS was included as part of the FCC-I Survey. eHEALS comprises 8 items that measure consumers’ perceived knowledge about how to find, use, and evaluate Internet-based health information to make informed health decisions. Response options are based on a 5-point Likert-type scale that ranges from 1 (strongly disagree) to 5 (strongly agree), with total summed eHealth literacy scores ranging from 8 (lowest possible eHealth literacy) to 40 (highest possible eHealth literacy).

### Data Analysis

An exploratory structural equation modeling (E-SEM) approach [41] using the weighted least squares and adjusted means and variances (WLSMV) estimator examined the model fit of eHEALS scores with 1-, 2-, and 3-factor structures. This model uses an exploratory factor analysis measurement model and applies a structural equation model to describe (1) which items significantly load onto the extracted factor(s); (2) the dimensionality or number of factors (or subscales) produced; and (3) the relationships between factors (if more than 1 factor is extracted). The following global model fit indices provided evidence of good model fit [42]: (1) root mean square error of approximation (RMSEA) value close to .06; (2) comparative fit index (CFI) value >.95; (3) Tucker-Lewis index (TLI) value >.95; and (4) nonstatistically significant chi-square test. We evaluated factor loadings of each item for statistical significance (*P*<.05) and computed fit indices for all 3 factor structures to determine the best overall model fit. We used Mplus v7.3 (Muthén & Muthén; [43]) to conduct all E-SEM analyses.

Following E-SEM analyses, we used the partial credit model (PCM), an IRT analysis [44,45], to explore the internal structure of the self-reported polytomous (ie, more than 2 possible response options) eHEALS data. This analysis was appropriate given that the final sample size (N=283) was over 200 cases and greater than 10 times the number of eHEALS items (ie, 8) [46,47]. PCM constrains item discrimination, or the strength (slope) of the relationship between responses and a latent trait. This provides important information on which response options have the greatest probability of being answered at a particular theta (ie, a person’s latent trait score) level on the latent continuum. Information from PCM analyses helps to evaluate stability across items, which reduces the potential for item bias [48,49]. Allowing step variability to vary across items provides useful information about the range of difficulties measured in a scale, including whether differences in step difficulties exist across items. RStudio’s eRm software package version 0.15-7 (R Foundation; [50]) computed all PCM estimates.

Finally, Linacre’s guidelines [46] for optimizing rating scales under IRT assumptions informed item fit analyses that calculated step difficulties of each response option. Optimized rating scales have threshold values (ie, relative difficulties to advance from one response option to the other) that increase across the theta continuum, which helps confirm that higher response options coincide with greater ability levels. Relative difficulties across response options helped to determine how precisely each eHEALS item was measured on the latent continuum. Values for each item that advanced less than 1.4 logits indicated a lack of variability across response categories, whereas values advancing more than 5.0 logits indicated extremely high variability, or low precision, between response categories.

Infit and outfit mean square (MSQ) and *t* statistics determined the level of noise or randomness in item response options. For outfit MSQ values, any value greater than 1.5 indicates unpredictable random error, whereas a value less than 1.0 indicates a degree of overpredictability and nonrandom error. Values less than 0.5 are interpreted as troublesome for overfit. For outfit *t* statistics, a value greater than 2.0 indicates underfit and less than –2.0 indicates overfit [50]. Measurement stability, which describes adequate item placement across the latent continuum, is determined based on adequate item reliability (>.80) and satisfactory item separability (>2.0) [51].

## Results

### Participant Characteristics

As reported by Tennant and colleagues [14], the mean age of respondents was 67.46 years (SD 9.98 years). Most respondents were white (n=252, 89.1%) and non-Hispanic (n=264, 93.3%). A little over half identified as being male (n=155, 54.8%). Over three-quarters of the sample (n=215, 75.9%) reported at least some college-level education, and over half (n=138, 60.4%) reported earning more than US $50,000 per year. Additionally, nearly three-quarters of respondents reported their health as being “good” (n=72, 25.1%), “very good” (n=103, 36.4%), or “excellent” (n=62, 21.9%). A little more than one-third of respondents reported accessing social media (n=101, 35.7%) to locate or share health information.

### Descriptive eHEALS Scores

Total eHEALS scores ranged from 11 to 40 (mean 29.05, SD 5.75). Table 1 presents the mean (SD) score for the response to each item. Internal consistency estimates of eHEALS data collected in this study were relatively high (Cronbach alpha=.91).

### Exploratory Structural Equation Modeling Analyses

Table 2 lists global model fit statistics and factor loadings for models fitting 1, 2, and 3 factors.

#### E-SEM Model 1 (1 Factor)

Only the 1-factor eHEALS structure had an eigenvalue greater than 1 (eigenvalue = 5.55). Despite high CFI and TLI values (.96 and .94, respectively), the RMSEA value, .24, exceeded the recommended value around .06 (Table 3). This high RMSEA value suggested poor structural fit of eHEALS in a unidimensional model.

**Table 1:** Mean (SD) eHealth Literacy Scale (eHEALS) scores rated on a 5-point Likert-type scale^a^.

eHEALS items		Mean	SD
E1.	I know what health resources are available on the Internet.	3.61	0.91
E2.	I know where to find helpful health resources on the Internet.	3.76	0.86
E3.	I know how to use the health information I find on the Internet to help me.	3.81	0.85
E4.	I know how to find helpful health resources on the Internet.	3.80	0.86
E5.	I have the skills I need to evaluate the health resources I find on the Internet.	3.72	0.93
E6.	I know how to use the Internet to answer my questions about health.	3.82	0.88
E7.	I can tell high quality health resources from low quality health resources on the Internet.	3.35	1.06
E8.	I feel confident in using information from the Internet to make health decisions.	3.19	1.09

^a^Scored from 1=strongly disagree to 5=strongly agree, where 1 indicates low confidence and 5 indicates high confidence.

**Table 2:** Factor loadings of the eHealth Literacy Scale (eHEALS) by dimension among adults 50 years of age and older surveyed by telephone (N=283).

eHEALS items		1 Factor		2 Factors				3 Factors					
		1	*P* value	1	*P* value	2	*P* value	1	*P* value	2	*P* value	3	*P* value
E1.	I know what health resources are available on the Internet.	0.71	<.05	0.73	<.05	0.05	NS^a^	0.71	<.05	–0.00	NS	0.21	<.05
E2.	I know where to find helpful health resources on the Internet.	0.89	<.05	1.01	<.05	–0.01	NS	0.82	<.05	0.24	<.05	0.00	NS
E3.	I know how to find helpful health resources on the Internet.	0.94	<.05	0.58	<.05	0.41	<.05	0.51	<.05	0.55	<.05	–0.02	NS
E4.	I know how to use the Internet to answer my questions about health.	0.85	<.05	0.01	NS	0.88	<.05	0.02	NS	0.8	<.05	0.03	NS
E5.	I know how to use the health information I find on the Internet to help me.	0.89	<.05	0.03	NS	0.89	<.05	0.01	NS	0.93	<.05	0.00	NS
E6.	I have the skills I need to evaluate the health resources I find on the Internet.	0.82	<.05	–0.15	<.05	0.97	<.05	–0.03	NS	0.59	<.05	0.37	<.05
E7.	I can tell high quality health resources from low quality health resources on the Internet.	0.75	<.05	–0.03	NS	0.79	<.05	0.15	NS	0.00	NS	0.88	<.05
E8.	I feel confident in using information from the Internet to make health decisions.	0.72	<.05	0.03	NS	0.72	<.05	0.10	NS	0.45	<.05	0.30	<.05

^a^NS: not statistically significant at *P*<.05 alpha level.

**Table 3:** Global model fit indices.

Indices	1 Factor	2 Factors	3 Factors
RMSEA^a^ (90% CI)	.24 (.21-.26)	.15 (.13-.18)	.07 (.02-.11)
Comparative fit index	.96	.99	1.0
Tucker-Lewis index	.94	.98	1.0
Chi-square test, *P* value	<.001	<.001	<.001
Eigenvalue	5.55	0.83	0.53

^a^RMSEA: root mean square error of approximation.

#### E-SEM Model 2 (2 Factors)

Global model fit indices improved in the 2-factor model (Table 3). CFI and TLI fit statistics improved to .99 and .98 respectively, while the RMSEA value decreased to .15. Even though RMSEA decreased in the 2-factor model, it remained over .07, which suggests poor global model fit. In the 2-factor model, eHEALS items 1 to 3 loaded onto factor 1, while items 4 to 8 loaded onto factor 2. Interestingly, item 3 appeared to have 2 relatively high (and statistically significant) factor loadings on both factors (factor 1=0.58; factor 2=0.41). However, it should be noted that these 2 factors were both highly correlated (*r*=.71, *P*<.01).

#### E-SEM Model 3 (3 Factors)

For the 3-factor model, global model fit indices were near the acceptable range (Table 3). CFI and TLI both improved to 1.0 and RMSEA decreased to .07. While the chi-square test of model fit remained nonsignificant, this statistic is sensitive to sample size and thus should be interpreted with caution [52]. Items 1 and 2 loaded onto factor 1, while item 3 (“I know how to use the health information I find on the Internet to help me”) significantly loaded onto both factors 1 (λ=.51) and 2 (λ=.55), making its assignment to 1 unique factor unclear. Similarly, we found that item 6 (“I know how to use the Internet to answer my questions about health”) loaded onto factors 2 (λ=.59) and 3 (λ=.37), as did item 8 (“I feel confident in using information from the Internet to make health decisions”; factor 2: λ=.45; factor 3: λ=.30). In the 3-factor model, we also found statistically significant correlations between factors 1 and 2 (*r*=.58), and between factors 2 and 3 (*r*=.65). Factors 1 and 3 were also significantly correlated, albeit to a lesser degree (*r*=.36).

### Partial Credit Model Analyses

The item reliability of eHEALS scores in this sample was estimated at .92 (observed variance=4.58), while the item separation index was 11.31. Both values were indicative of high reliability and stability across the latent continuum.

Table 4 shows that Linacre’s assumption of monotonicity was satisfied, with thresholds (ie, relative difficulty advancing from response options) increasing across the theta continuum, as demonstrated in the item characteristic curves and reported threshold values, confirming that greater eHealth literacy coincided with higher response options. However, not all step difficulties advanced from 1.4 to 5 logits. Relative difficulty moving from “strongly disagree” to “disagree” for almost all items was less than 1.4 logits, except for eHEALS item 8 (“I feel confident in using information from the Internet to make health decisions”), where it was 1.96. Relative difficulty moving from “agree” to “strongly agree” was within the acceptable range for all items (ie, all below 5.0 logits), but they were quite large as compared with advances in the relative difficulty for thresholds 1 (“strongly disagree” to “disagree”), 2 (“disagree” to “neutral”), and 3 (“neutral” to “agree”) [46].

Table 5 shows that all outfit MSQ values were <2.0 yet closer to 1.0, which suggested an optimal degree of randomness in responses to eHEALS items. However, the outfit MSQ values for items 2 to 5 fell well below 1.0, suggesting some level of overpredictability (ie, respondents with a particular eHealth literacy level were responding to items 2 to 5 using similar response options). Subsequently, we noted that infit *t* statistics for items 2 to 5 were all below –2.0, which is outside of the acceptable range of –2 to 2.

**Table 4:** Threshold^a^ values of response options for 8-item eHealth Literacy Scale (eHEALS).

eHEALS items	Item difficulty	Threshold 1	Threshold 2	Threshold 3	Threshold 4
E1	0.85	–1.63	–0.77	1.19	4.63
E2	0.69	–1.45	–0.84	0.36	4.68
E3	0.68	–1.35	–0.49	–0.09	4.65
E4	0.66	–1.15	–0.47	–0.19	4.47
E5	0.61	–1.53	–0.53	–0.07	4.58
E6	0.86	–1.28	–0.09	0.10	4.70
E7	1.55	–0.86	0.34	1.38	5.36
E8	1.76	–1.12	0.84	1.83	5.49

^a^Thresholds for response options on the 5-point Likert-type scale: 1 (from “strongly disagree” to “disagree”), 2 (from “disagree” to “neutral”), and 3 (from “neutral” to “agree”).

**Table 5:** Infit and outfit mean square (MSQ), and infit and outfit *t* statistics for eHealth Literacy Scale (eHEALS) items.

eHEALS items	*P* value	Infit MSQ	Outfit MSQ	Infit *t* statistic	Outfit *t* statistic
E1	.04	1.16	1.16	1.65	1.16
E2	>.99	0.70	0.70	–2.09	0.80
E3	>.99	0.54	0.54	–3.74	0.64
E4	>.99	0.61	0.61	–2.56	0.74
E5	>.99	0.60	0.60	–3.55	0.66
E6	.95	0.86	0.86	–1.30	0.87
E7	.32	1.03	1.03	0.47	1.04
E8	.22	1.06	1.06	0.55	1.05

**Figure 1:**
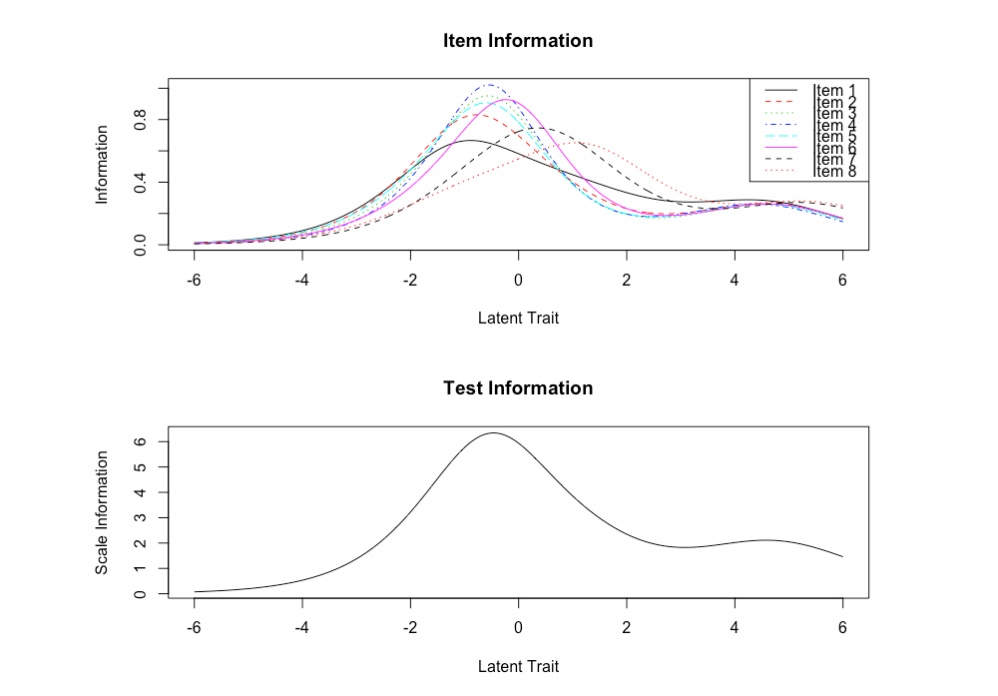
Item and test information function curves for eHealth Literacy Scale (eHEALS).

Figure 1 depicts item and test information functions. The test information curve shows a high degree of information with minimal standard measurement error around theta levels –2 to 2 on the latent continuum. The test information curve shows that eHEALS provides some degree of information for participants at the higher end of the latent continuum, but reliability and validity evidence for this level of information is likely unstable. Moreover, the test information function is positively skewed, rather than bell shaped. This result indicates that eHEALS items may capture more information about eHealth literacy among participants who place higher on the latent continuum (ie, those with high eHealth literacy) than among those at the lower end of the continuum (ie, those with low eHealth literacy).

Item information curves showed that all eHEALS items followed a similar curvature pattern, yet the peak of most item curves (greatest amount of information) were plotted at different points on the latent continuum. Despite test information functions that were positively skewed, item information curves suggested that each eHEALS item made important contributions to the complete measure of eHealth literacy. Interestingly, information obtained from items 2 to 5 did not vary across different points on the latent continuum. Therefore, items 2 to 5 may produce a similar amount of information at each point on the latent continuum.

## Discussion

This measurement study was the first, to our knowledge, to provide evidence for the factor structure and dimensionality of eHEALS when administered to older adults over the telephone. Overall, results from E-SEM and PCM analyses support that use of eHEALS as a reliable measure of eHealth literacy produces a sufficient degree of internal structure reliability evidence when administered to older adults using telephone-based survey methods. Despite several poor-fitting items in this brief 8-item instrument, global model fit indices produced by E-SEM analyses suggest the eHEALS has the potential for 3 factors (or subscales) when measuring the latent construct of eHealth Literacy among older adults. However, 2 of these 3 factors were highly correlated with one another, providing additional evidence to support an overarching unidimensional structure of eHEALS data when collected in an older adult population.

Similar to this study, whose findings suggesting that a 3-factor solution is promising in the older adult population, the study of Sudbury-Riley et al [32] found 3 underlying factors in eHEALS data collected from baby boomers located in 3 different countries (United States, United Kingdom, and New Zealand). The 3 factors identified by those authors were awareness and learning about what online resources are available and where they are located (items 1-2), skills and behaviors needed to access Internet-based health resources (items 3-5), and the self-belief in one’s ability to evaluate online health content once accessed (items 6-8). Further, Sudbury-Riley and colleagues suggested that these 3 factors reflected social cognitive theory’s explanation of a triadic reciprocal causation among 3 dimensions (personal factors, behavioral factors, and environmental factors) that influence behavior change [53]. Data from our study produced acceptable fit indices for assigning eHEALS items to these 3 factors; however, item 3 (“I know how to use the health information I find on the Internet to help me”), item 6 (“I know how to use the Internet to answer my questions about health”), and item 8 (“I feel confident in using information from the Internet to make health decisions”) loaded onto multiple factors, which made it difficult to assign these particular items to the 3 unique eHEALS subscales. Moreover, these 3 factors showed moderate to high correlations with one another, which supports the reciprocity described in social cognitive theory. The relationship between personal motivations for health information seeking and an individual’s perceived capability to use digital technologies can be affected by online environments with socially persuasive forms of media. Since social cognitive theory was the theoretical foundation used during the original development of eHEALS [27], future research should investigate how eHEALS items map to the main theoretical constructs of social cognitive theory.

Linacre’s [46] guidelines for optimizing rating scales were satisfied regarding item fit of eHEALS data in this study. Even with constrained item discrimination, item characteristic curves showed that each response option had the highest probability of selection at a particular point on the latent continuum. This suggests that the rating scale is functioning as intended, where older adults higher on the eHealth literacy continuum demonstrate the greatest probability of selecting response option “agree” or “strongly agree,” and individuals scoring lower on the eHealth literacy continuum have the greatest probability of selection response option “disagree” or “strongly disagree.” This finding is similar to results reported in previous research exploring the internal structure of eHEALS [28,30], which noted that data produced by eHEALS among older adults showed evidence of monotonicity.

Step difficulties also advanced within acceptable standards [46] across the latent continuum for each eHEALS item. Tests of the internal structure of each item showed that step difficulties advancing across response options 1 to 3 were located close to one another on the latent continuum. In contrast, the relative difficulty of advancing from “agree” to “strongly agree” was located further away from the threshold, suggesting relative difficulty of advancing from “neutral” to “agree” response options. To capture this “dead zone” between these 2 response option thresholds, future research should consider analyzing the effects of adding more response options to each eHEALS item.

Given that the 3 factors identified in this study showed moderately strong correlations with one another and the 1-factor model showed adequate fit, we conducted item fit analyses using PCM analyses. Like in the work of Diviani et al [34], who administered the Italian version of eHEALS in young to middle-aged adults, in this study the level of random error in eHEALS responses from older adults was within the acceptable range. However, parametric IRT analyses did reveal that items 2 to 5, which assessed knowledge of using the Internet to access and use health information, showed a minor degree of overpredictability and random error. This minor level of overpredictability on eHEALS items 2 to 5 was less evident in the Diviani et al [34] study’s younger sample, although eHEALS items 1 through 5 in their study did show outfit MSQ values of less than 1. While data that are potentially overfit do not present a substantial threat to measurement validity [46], eHEALS items asking about finding knowledge and using Internet-based health information may be redundant, with the potential to (1) violate the assumption of local independence, (2) overestimate the reliability of eHEALS, and (3) underestimate the standard error of eHEALS measurements [54]. It is important to note that the negative impact of overfit in both studies is likely minimal, given relatively minor deviations from acceptable values [46]. However, in both Diviani et al [34] and our study of older adults, eHEALS items 2 and 4 had standardized infit *t* statistics less than the lower end of the acceptable range (less than –2.0). Conducting think-aloud cognitive interviews with respondents while they complete the eHEALS should provide much-needed information regarding whether older adults perceive different eHEALS items to be asking the same questions.

### Limitations

There are several limitations to note in this study. First, this was a cross-sectional study and, therefore, we were unable to compute test-retest reliability or predictive validity estimates. Second, our analyses used telephone survey data with a very low overall response rate (7.4%), resulting in the possibility of nonresponse bias. Third, comparative measures of model fit did not inform decisions regarding the optimal internal structure of eHEALS data collected in this study. Comparative fit measures such as the Akaike information criterion can only be estimated with maximum likelihood data extraction methods, which Mplus v7.3 does not allow for under the WLSMV estimator. We selected the WLSMV estimator to examine model fit in this study for several reasons: (1) WLSMV estimation compensates more effectively than the maximum likelihood estimation for bias due to ordinal response options in the eHEALS, and (2) WLSMV estimation is less likely to produce unrealistic indices of overall model fit [55,56]. Therefore, we based conclusions regarding the internal structure of eHEALS data on noncriterion-based judgments made through interpretation of E-SEM and IRT analyses results.

Fourth, this study contacted participants through a landline sampling technique, which may have selectively excluded individuals who may only own a mobile phone. Although this telephone sampling method targeted older adults living in the state of Florida, this state is home to the greatest proportion (19.1%) of older adults in the United States [57].

Fifth, this study examined eHEALS responses derived from telephone administration of the survey, despite all participants reporting use of the Internet or email. Widespread adoption of the Internet and mobile phone technology has contributed to nonuse of landline telephones. We did not account for mobile devices and cellular telephones, which are increasingly being used by middle- to older-aged adults [58], in this sample. Partnering with community-engaged research programs or local community organizations to reach older adults via telephone may enhance recruitment efforts in this population traditionally underrepresented in health-related survey research.

### Conclusions

Assessing consumer comfort and self-efficacy in using technology to access online health resources can help identify skill gaps and gauge the likelihood that users will be successful when using the Internet to access relevant health information [23]. Results from this study suggest that administering eHEALS to older adults via telephone produces a reliable measure with scores that possess sufficient construct validity evidence. Specifically, results from this study support the previously reported unidimensionality of eHEALS scores. Among older adults, however, there is potential for additional underlying subscales to measure older adults’ confidence to locate, use, and evaluate online health information. As older Internet users continue to visit online support groups and discussion forums to find new information about health care perspectives and experiences, it will be important to consider modifying the original eHEALS to adequately measure online health information-seeking behaviors in older populations.

## Abbreviations

CFI: comparative fit index
eHEALS: eHealth Literacy Scale
E-SEM: exploratory structural equation modeling
F-CCI: Florida Consumer Confidence Index
IRT: item response theory
MSQ: mean square
PCM: partial credit model
RMSEA: root mean square error of approximation
TLI: Tucker-Lewis index
WLSMV: weighted least squares and adjusted means and variances

## References

[1] Jha A, Pandey J (2017). An empirical note on health information digital divide: a study of Indian patients. Int J Asian Bus Inf Manage.

[2] van Deursen AJAM, van Dijk JAGM (2011). Internet skills performance tests: are people ready for eHealth?. J Med Internet Res.

[3] Fox S, Duggan M (2013). Health online 2013.

[4] Zickuhr K (2010). Generations 2010.

[5] Benigeri M, Pluye P (2003). Shortcomings of health information on the Internet. Health Promot Int.

[6] Madathil KC, Rivera-Rodriguez AJ, Greenstein JS, Gramopadhye AK (2015). Healthcare information on YouTube: a systematic review. Health Informatics J.

[7] Neter E, Brainin E, Baron-Epel O (2015). The dimensionality of health literacy and eHealth literacy. Euro Health Psych.

[8] Chaudhuri S, Le T, White C, Thompson H, Demiris G (2013). Examining health information-seeking behaviors of older adults. Comput Inform Nurs.

[9] LeRouge C, Van SC, Seale D, Wright K (2014). Baby boomers' adoption of consumer health technologies: survey on readiness and barriers. J Med Internet Res.

[10] Anderson M, Perrin A (2017). Tech adoption climbs among older adults.

[11] Amante DJ, Hogan TP, Pagoto SL, English TM, Lapane KL (2015). Access to care and use of the Internet to search for health information: results from the US National Health Interview Survey. J Med Internet Res.

[12] King DE, Matheson E, Chirina S, Shankar A, Broman-Fulks J (2013). The status of baby boomers' health in the United States: the healthiest generation?. JAMA Intern Med.

[13] Stellefson M, Chaney B, Barry AE, Chavarria E, Tennant B, Walsh-Childers K, Sriram PS, Zagora J (2013). Web 2.0 chronic disease self-management for older adults: a systematic review. J Med Internet Res.

[14] Tennant B, Stellefson M, Dodd V, Chaney B, Chaney D, Paige S, Alber J (2015). eHealth literacy and Web 2.0 health information seeking behaviors among baby boomers and older adults. J Med Internet Res.

[15] Zhang S, Grenhart WC, McLaughlin AC, Allaire JC (2017). Predicting computer proficiency in older adults. Comput Hum Behav.

[16] Jacobs W, Amuta AO, Jeon KC (2017). Health information seeking in the digital age: an analysis of health information seeking behavior among US adults. Cogent Soc Sci.

[17] Kobayashi LC, Wardle J, von Wagner C (2015). Internet use, social engagement and health literacy decline during ageing in a longitudinal cohort of older English adults. J Epidemiol Community Health.

[18] Smith SG, O'Conor R, Curtis LM, Waite K, Deary IJ, Paasche-Orlow M, Wolf MS (2015). Low health literacy predicts decline in physical function among older adults: findings from the LitCog cohort study. J Epidemiol Community Health.

[19] Li N, Orrange S, Kravitz RL, Bell RA (2014). Reasons for and predictors of patients' online health information seeking following a medical appointment. Fam Pract.

[20] Tan SS, Goonawardene N (2017). Internet health information seeking and the patient-physician relationship: a systematic review. J Med Internet Res.

[21] Bhandari N, Shi Y, Jung K (2014). Seeking health information online: does limited healthcare access matter?. J Am Med Inform Assoc.

[22] Lee ST, Lin J (2016). A self-determination perspective on online health information seeking: the Internet vs. face-to-face office visits with physicians. J Health Commun.

[23] Norman CD, Skinner HA (2006). eHealth literacy: essential skills for consumer health in a networked world. J Med Internet Res.

[24] Hsu W, Chiang C, Yang S (2014). The effect of individual factors on health behaviors among college students: the mediating effects of eHealth literacy. J Med Internet Res.

[25] Mitsutake S, Shibata A, Ishii K, Oka K (2016). Associations of eHealth literacy with health behavior among adult Internet users. J Med Internet Res.

[26] Milne RA, Puts MTE, Papadakos J, Le LW, Milne VC, Hope AJ, Catton P, Giuliani ME (2015). Predictors of high eHealth literacy in primary lung cancer survivors. J Cancer Educ.

[27] Norman CD, Skinner HA (2006). eHEALS: the eHealth Literacy Scale. J Med Internet Res.

[28] Nguyen J, Moorhouse M, Curbow B, Christie J, Walsh-Childers K, Islam S (2016). Construct validity of the eHealth literacy scale (eHEALS) among two adult populations: a Rasch analysis. JMIR Public Health Surveill.

[29] Chung S, Nahm E (2015). Testing reliability and validity of the eHealth Literacy Scale (eHEALS) for older adults recruited online. Comput Inform Nurs.

[30] Paige SR, Krieger JL, Stellefson M, Alber JM (2017). eHealth literacy in chronic disease patients: an item response theory analysis of the eHealth literacy scale (eHEALS). Patient Educ Couns.

[31] Paige SR, Krieger JL, Stellefson ML (2017). The influence of eHealth literacy on perceived trust in online health communication channels and sources. J Health Commun.

[32] Sudbury-Riley L, FitzPatrick M, Schulz PJ (2017). Exploring the measurement properties of the eHealth literacy scale (eHEALS) among baby boomers: a multinational test of measurement invariance. J Med Internet Res.

[33] Soellner R, Huber S, Reder M (2014). The concept of eHealth literacy and its measurement. J Media Psychol.

[34] Diviani N, Dima AL, Schulz PJ (2017). A psychometric analysis of the Italian version of the eHealth Literacy Scale using item response and classical test theory methods. J Med Internet Res.

[35] Bowling A (2005). Mode of questionnaire administration can have serious effects on data quality. J Public Health (Oxf).

[36] Wyatt JC (2000). When to use web-based surveys. J Am Med Inform Assoc.

[37] Dillman D (2007). Mail and Internet Surveys: The Tailored Design Method. 2nd edition.

[38] Neter E, Brainin E (2017). Perceived and performed ehealth literacy: survey and simulated performance test. JMIR Hum Factors.

[39] University of Florida Survey Research Center (2014). Telephone survey methods.

[40] Watkins I, Xie B (2014). eHealth literacy interventions for older adults: a systematic review of the literature. J Med Internet Res.

[41] Asparouhov T, Muthén B (2009). Exploratory structural equation modeling. Struct Equ Model.

[42] Hu L, Bentler PM (1999). Cutoff criteria for fit indexes in covariance structure analysis: conventional criteria versus new alternatives. Struct Equ Model.

[43] Muthén LK, Muthén BO (2012). Mplus: statistical analysis with latent variables. Users guide. 7th edition.

[44] Masters GN (1982). A rasch model for partial credit scoring. Psychometrika.

[45] Masters G, Hambleton RK, van der Linden WJ (1996). The partial credit model. Handbook of Modern Item Response Theory.

[46] Linacre JM (2002). Optimizing rating scale category effectiveness. J Appl Meas.

[47] MacCallum RC, Widaman KF, Zhang S, Hong S (1999). Sample size in factor analysis. Psychol Methods.

[48] Embertson SE, Reise SP (2000). Item Response Theory for Psychologists. Multivariate Applications Book Series.

[49] Luo G (2005). The relationship between the rating scale and partial credit models and the implication of disordered thresholds of the Rasch models for polytomous responses. J Appl Meas.

[50] Mair P, Hatzinger R, Maier MJ, Rusch T (2016). Package 'eRm'.

[51] Fox CM, Jones JA (1998). Uses of Rasch modeling in counseling psychology research. J Couns Psychol.

[52] Barrett P (2007). Structural equation modelling: adjudging model fit. Pers Individ Differ.

[53] Bandura A (1986). Social Foundation of Thought and Action: A Social Cognitive Theory.

[54] Beglar D (2009). A Rasch-based validation of the Vocabulary Size Test. Lang Test.

[55] Beauducel A, Herzberg PY (2006). On the performance of maximum likelihood versus means and variance adjusted weighted least squares estimation in CFA. Struct Equ Model.

[56] Olsson UH, Foss T, Troye SV, Howell RD (2000). The performance of ML, GLS, and WLS estimation in structural equation modeling under conditions of misspecification and nonnormality. Struct Equ Model.

[57] Kent L (2015). Where do the oldest Americans live?.

[58] Anderson M, Perrin A (2017). Tech adoption climbs among older adults.

